# Comprehensive Analysis of Circular RNAs in Endothelial Cells

**DOI:** 10.3390/ijms241210025

**Published:** 2023-06-12

**Authors:** Sabina Lichołai, Dorota Studzińska, Hanna Plutecka, Tomasz Gubała, Marek Sanak

**Affiliations:** 1 Division of Molecular Biology and Clinical Genetics, Faculty of Medicine, Jagiellonian University Medical College, 31-066 Krakow, Polandmarek.sanak@uj.edu.pl (M.S.); 2 Department of Intensive Care and Perioperative Medicine, Faculty of Medicine, Jagiellonian University Medical College, 31-901 Krakow, Poland; 3 Sano—Centre for Computational Medicine, 30-072 Krakow, Poland

**Keywords:** circular RNAs, endothelial cells, epigenetic regulation

## Abstract

Non-coding RNAs constitute a heterogeneous group of molecules that lack the ability to encode proteins but retain the potential ability to influence cellular processes through a regulatory mechanism. Of these proteins, microRNAs, long non-coding RNAs, and more recently, circular RNAs have been the most extensively described. However, it is not entirely clear how these molecules interact with each other. For circular RNAs, the basics of their biogenesis and properties are also lacking. Therefore, in this study we performed a comprehensive analysis of circular RNAs in relation to endothelial cells. We identified the pool of circular RNAs present in the endothelium and showed their spectrum and expression across the genome. Using different computational strategies, we proposed approaches to search for potentially functional molecules. In addition, using data from an in vitro model that mimics conditions in the endothelium of an aortic aneurysm, we demonstrated altered expression levels of circRNAs mediated by microRNAs.

## 1. Introduction

Non-coding RNAs constitute a heterogeneous group of molecules that retain the ability to regulate biological processes despite mostly not being translated into proteins. Until recently, the main classification of these molecules was based on their length, with microRNAs and long non-coding RNAs being the dominant subgroups. More recent evidence has shown that there is another distinction that is equally valid. It has been shown that, in addition to the classic linear transcripts, there is a large group of circular transcripts with functions that are not yet fully understood.

Non-coding RNAs play a key role in epigenetic modifications; thus, they can regulate expression at the level of genes or even whole chromosomes, affecting numerous cellular processes. The exact processes and the exact biogenesis of the individual molecules are still not completely clear.

Circular RNAs (CircRNAs) are single-stranded, covalently closed RNA molecules [1]. Important advances have been accomplished regarding knowledge about their biogenesis, regulation, localization, degradation, and modification [2]. They appear to originate from the same genes (host genes) as linear transcripts but in a different way and under conditions that are not completely elucidated. CircRNAs are thought to exert their biological functions by acting as transcriptional regulators, e.g., microRNA (miR) sponges. Some evidence has pointed to a potential ability to be a template for proteins, making the whole group of these molecules questionable as belonging to the non-coding RNAs [3]. Moreover, emerging evidence has revealed that a group of circRNAs can serve as protein decoys, scaffolds, and recruiters [4]. However, the existing research on circRNA-protein interactions is quite limited. The regulation of circRNA expression has also not been fully elucidated. It appears that at least several mechanisms may be involved in this process, and the roles of both cis- and trans-elements have been highlighted. Among the trans-regulatory elements, the involvement of RBPs, such as MBL or QKI, has been demonstrated. Another regulation process is associated with NOVA2, which appears to regulate circular RNA biogenesis in neurons [5].

MicroRNAs, on the other hand, are a group of short linear non-coding RNAs about 22 nucleotides long. Their biogenesis and functions are much better understood. They influence virtually every process in the cell by silencing gene expression without changing the DNA structure. They bind to linear mRNA transcripts through their target sites, usually located within the 3′ untranslated region (UTR). Depending on whether there is complete or only partial complementarity between the microRNAs and their target sites, translation inhibition is preceded by degradation of the entire transcript or not. Because a single gene can be regulated by multiple microRNAs, and each microRNA regulates more than one gene, these interactions are pleiotropic and form a complex regulatory network. Recent studies have indicated that other non-coding RNAs are also involved, including circRNAs.

One of the most interesting models for studying such complex relationships is endothelial cells. These cells line the entire inner surface of the vessel lumen and play an important role in maintaining vascular homeostasis. The heterogeneity of endothelial cells has been described, but it is still possible to identify many of the features that they have in common. For example, they express CD31 and von Willebrand factor. On the other hand, endothelial cell phenotypes vary depending on organ, tissue, or vascular segment in which they originate. For instance, in terms of structure, the endothelium cells lining an artery or vein align with the longitudinal axis of blood flow [6]. Additionally, endothelial cell dysfunction may be associated with the pathogenesis of various vascular diseases, including atherosclerosis or abdominal aortic aneurysms. In one of our previous works, we showed that the levels of miR-191, miR-126, and miR-21-5p are significantly different between patients with abdominal aortic aneurysms and healthy controls [7]. Studies in recent years have indicated a complex network of relationships between different fractions of noncoding RNAs that interact with each other. Therefore, in this study, we aimed to perform a comprehensive analysis of circular RNAs in relation to endothelial cells. We used iCell Endothelial Cells, which are purified human endothelial cells derived from induced pluripotent stem cells. iCell Endothelial Cells demonstrate characteristic gene and protein expression (such as CD31, CD105, CD144, ZO-1, and von Willebrand factor) and endothelial cell functions (such as tubular network formation, acetylated LDL uptake, barrier function, and wound healing). We started by defining the basal transcriptome of endothelial cells, including circular RNAs. We performed a characterization of the molecules obtained, and using different computational approaches, we investigated how to identify the molecules with the highest functional potential. Furthermore, using data from an in vitro model reproducing conditions characteristic of abdominal aortic aneurysms, we showed how the level of circular RNAs changes under the influence of microRNAs and proposed an approach based on the length/exon ratio to identify molecules with the highest functional potential.

## 2. Results

In this study, we used induced pluripotent stem cells differentiated into an endothelial phenotype, which were cultured under basal conditions as specified by the manufacturer’s protocol. This material was used for RNA isolation and construction of libraries for RNA sequencing, preceded by ribodepletion with subsequent transcriptome reconstruction. Characterization of the obtained circular RNAs is presented below.

### 2.1. Characterization of Circular RNAs in Endothelial Cells

The transcriptome reconstruction of endothelial cells yielded a total of 1068 unique circular RNA sequences. Of these sequences, 1055 were previously annotated in publicly available databases, and 1007 of the obtained transcripts originated from protein-coding genes. The number of circRNAs for which more than 50 reads was obtained was 17. Fourteen circRNAs were more abundant than their linear counterparts derived from the same gene. These results are summarized in Table 1, and a list of the 20 most abundant circRNAs is presented in the supplementary materials (Table S1).

Next, we compared the sequences obtained in the current study with publicly available circular RNA databases. Thirteen transcripts we found that were not previously annotated, and three of them were located within protein-coding genes. They are listed in Table 2.

**Figure 1:**
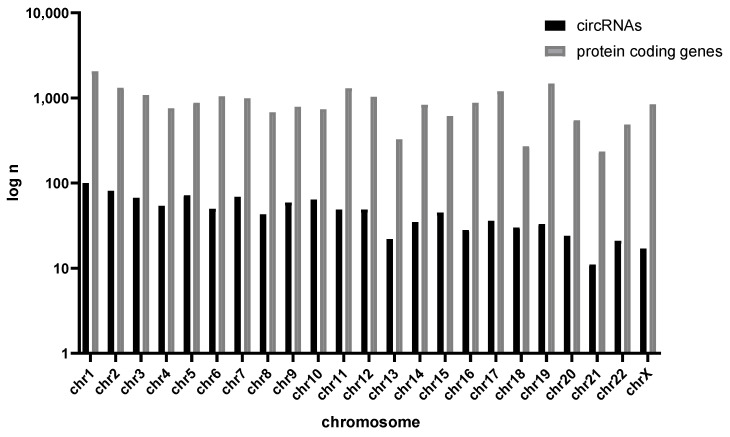
Number of circular RNAs across the genome accompanying the number of protein coding genes.

**Figure 2:**
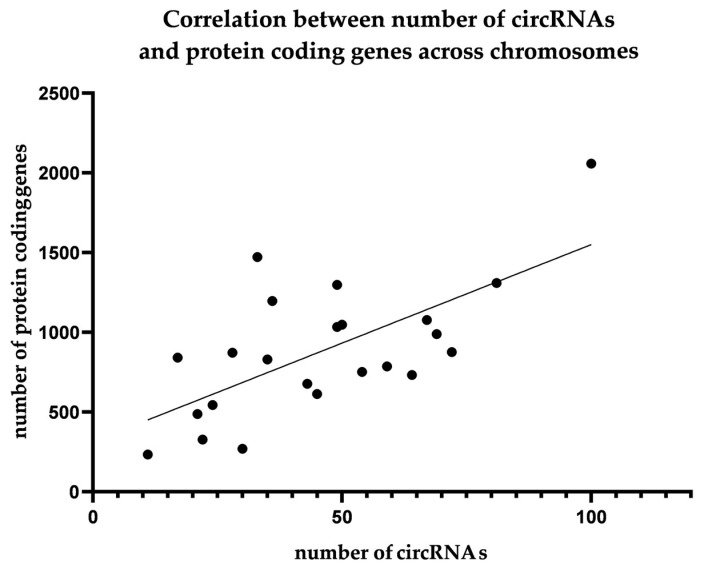
There is a moderate, positive correlation between the number of genes across chromosomes and the number of circular RNAs (r = 0.5871, *p* = 0.0032).

We also performed an analysis of the abundance of the obtained circular RNAs depending on the genomic location. For each chromosome, we determined the number of genes located on it and the number of circular RNAs derived from it. This analysis did not reveal any deviations from the expected distribution. Obtained results are shown as Figure 1 and Figure 2.

**Figure 3:**
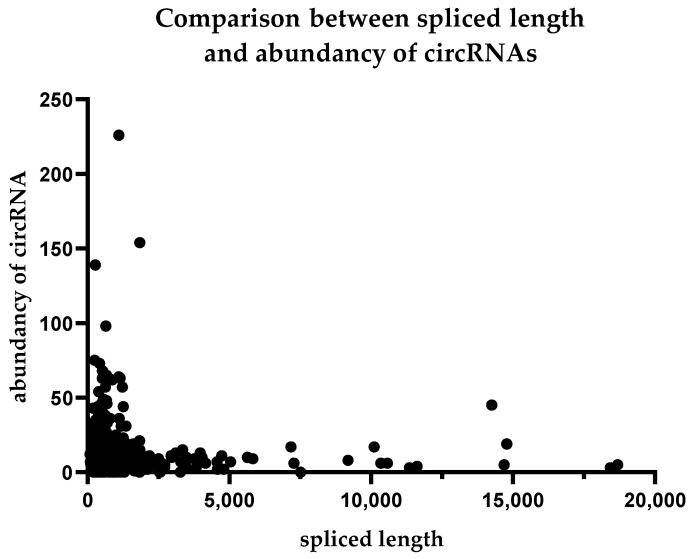
Comparison between the expression level of circular RNAs and their spliced length.

We also determined the spliced length distribution of individual circular RNAs. Endothelial cells were dominated by relatively short circular RNAs that were less than 5000 nucleotides in length when spliced. In contrast, we also found molecules with lengths exceeding 15,000 nucleotides. A comparison between the spliced length of circular RNAs and their abundance is shown in Figure 3.

Finally, we compared how many circular RNAs are formed from each gene. In most cases (587 genes) only one unique circular RNA is formed. For 126 genes, there were two alternative transcripts. For two genes (*CRIM1* and *PTK2*), we found as many as eight different circular RNAs. A summary of these results is presented in Table 3.

### 2.2. Comparison between Host Gene Structure and Abundance of circularRNA

**Figure 4:**
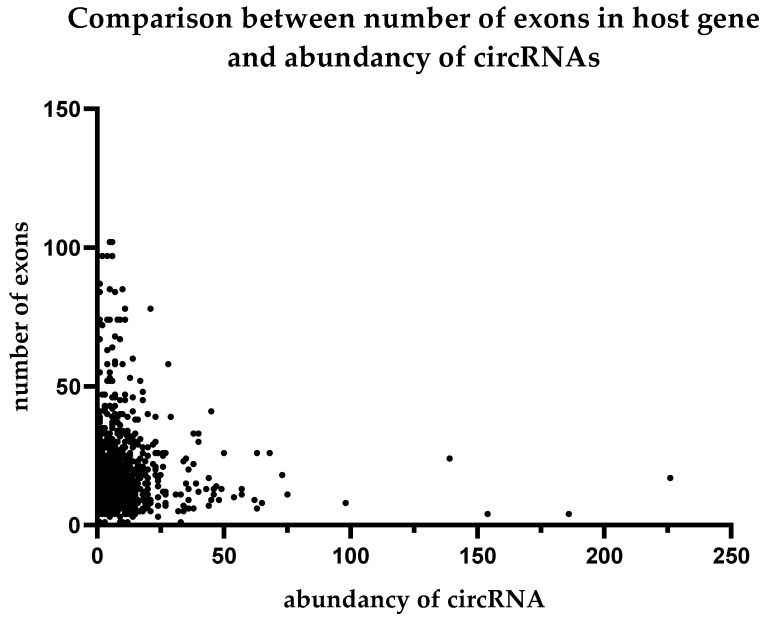
Comparison between the number of exoms in host gene and abundancy of circRNAs.

**Figure 5:**
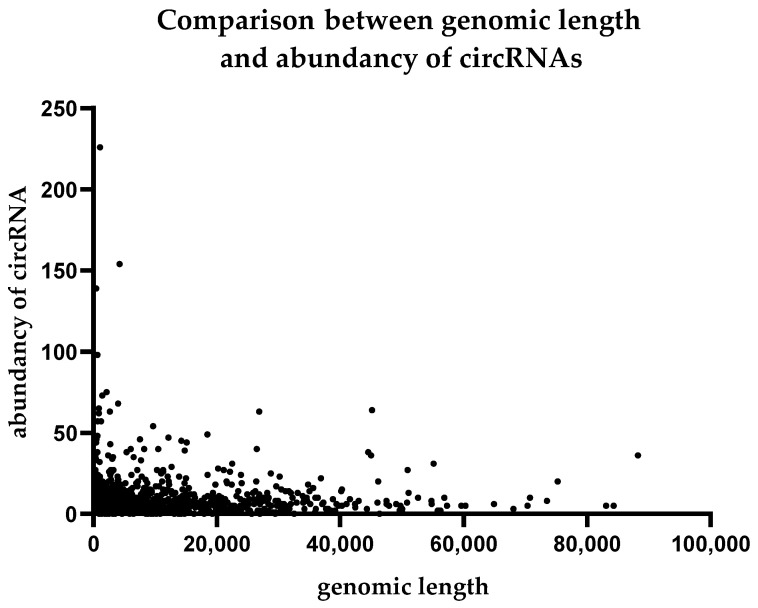
Comparison between genomic length and abundancy of circRNAs.

We performed a comparison between the expression levels of circular RNAs and the structures of their host genes. We did not find any correlation of circRNA abundance with genomic length or the exon number in the genes from which the circular RNAs formed. The results are summarized in Figure 4 and Figure 5.

### 2.3. Comparison between Expression Level of Circular RNA and Host Genes

**Figure 6:**
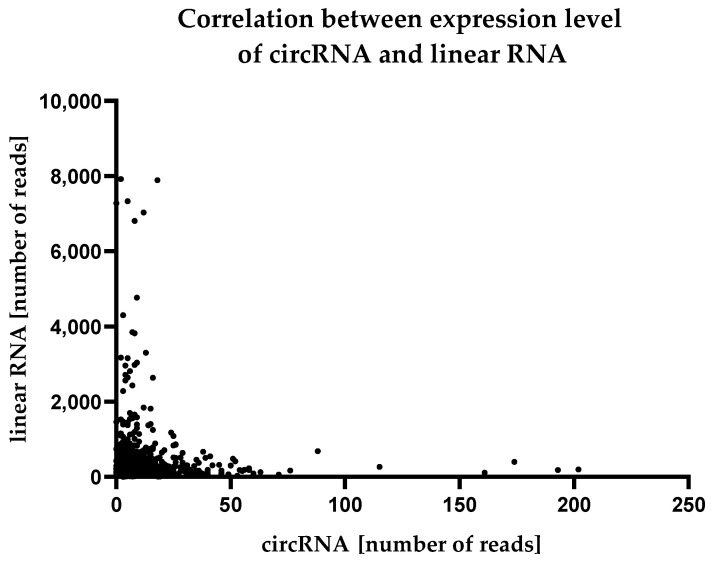
Comparison between the expression level of circular RNAs and mRNA encoded by the same host gene.

In the next step, we compared the abundancy of circRNAs in endothelial cells with the corresponding expression level of a linear mRNA encoded by the same host gene. Of the 1068 circRNA molecules, we found the vast majority (1030) were less abundant in the endothelial cells than linear RNA. For 24 molecules, the expression levels of circRNA and linear RNA were approximately equal. Fourteen circRNAs were at significantly higher levels than their linear counterparts. This distribution is shown in Figure 6.

A list of 14 genes for which the expression level of the linear version of mRNA was lower than that of circular RNA is shown in Table S2 (Supplementary Materials). According to the Reactome database, these host genes are associated with NOTCH signaling pathways. Additionally, platelet hemostasis and Ca^2+^ channels are involved.

### 2.4. Experimental Validation of Selected Circular RNAs Expressed Levels

The levels of a selected subset of 10 circular RNAs were determined by quantitative real-time PCR to confirm the results obtained by prediction using the bioinformatics algorithm. The circular RNA molecules were selected to reflect the range of possible expression in endothelial cells. The level of the most abundant has_circ_0000284 was more than 200 reads, whereas has_circ_0004087 was found in only a few copies. Specific primers were designed for each of the 10 selected molecules, and the expression level normalized to the 18S gene is shown in Table 4. We detected all selected circRNAs in native endothelial cells by qPCR. Moreover, there was a statistically significant, strong correlation between the expression levels measured by these two techniques (R = −0.8667, 0.0022, Spearman’s correlation).

### 2.5. Stimulation with microRNA Mimics

**Figure 7:**
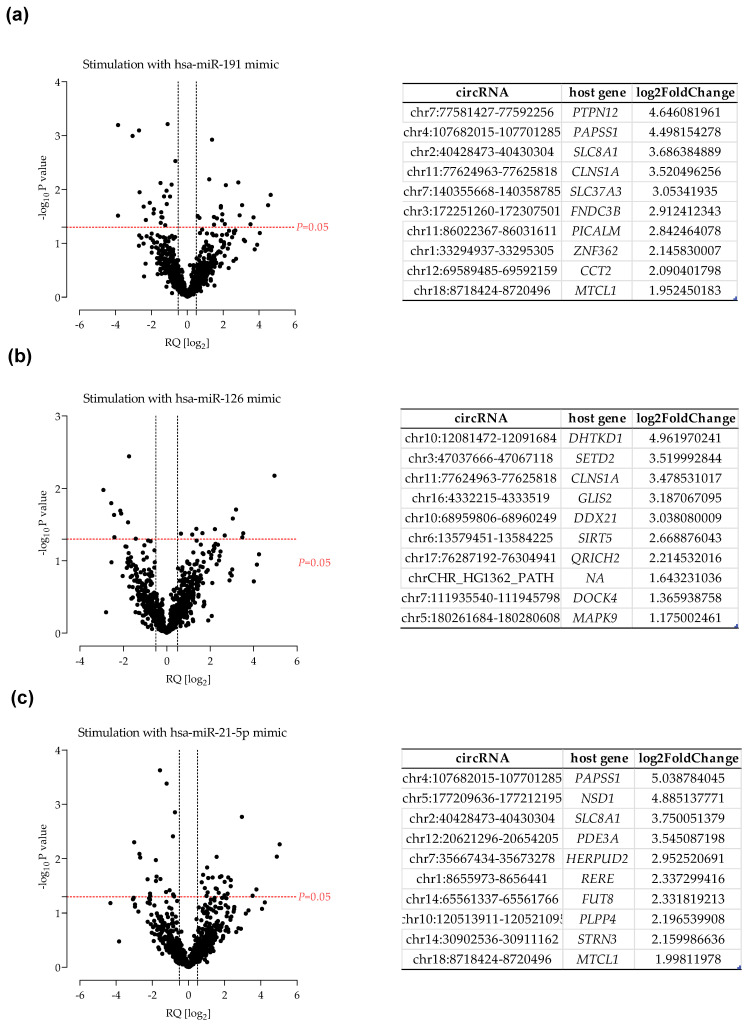
Differential expression of circular RNAs after stimulation with miR-191 (**a**), miR-126 (**b**), and miR-21 (**c**) mimics summarized as a volcano plot with associated 10 most upregulated molecules.

However, the function of circular RNAs in endothelial cell biology has not been fully elucidated, although some working hypothesis have been proposed. For example, it has been suggested that, by binding with microRNA, they may act as microRNA sponges and inhibit their function, thereby indirectly affecting gene expression levels. However, it is not clear whether changes in microRNA expression can induce changes in circular RNA levels and how the network of their interactions is established. Therefore, in the next step, we performed endothelial cell stimulation using microRNA mimics. We used a model of vessel wall dysfunction that we reported previously in the context of abdominal aortic aneurysms, in which we showed that the levels of three microRNAs (miR-191, miR-126, miR-21) induced changes identical to those observed in aneurysmal aortas. Cells were stimulated with microRNA-mimics, and isolated RNA was used to create libraries for RNA sequencing. The altered levels of circular RNAs are shown in Figure 7.

### 2.6. The Ratio of the Genomic Length to the Number of Exons in the Host Gene as an Indicator of Altered Circular RNA Expression

It is not entirely clear how circular RNA expression is regulated. Unlike linear forms of mRNA, the expression levels of which are subject to a variety of regulatory mechanisms, changes in circular RNA levels appear more chaotic. In the present study, we analyzed the expression level of circular RNA and compared it with the location or structure of host genes. Under microRNA stimulation, a specific pool of circular RNA molecules demonstrated altered expression. MicroRNA inhibits expression at the post-translational level and has its target sites on specific genes. However, there is no correlation or overrepresentation of changes in expression levels of circular RNAs encoded by the host genes on which target genes for specific microRNAs are located. The results are summarized in Table 5.

Interestingly, for four genes (*RAPGEF5*, *RICTOR*, *PKN1*, *GTDC1*), the derived circRNAs showed statistically significantly altered expression in each of the transfection variants by microRNAs, despite having no specific target sites.

In contrast, we were able to show that the greatest potential for overexpression of circular RNAs are molecules in which the ratio of host gene genomic length to the number of its exons is lower than the genome-wide average (Table 6). In the pool of these molecules, we observed an overrepresentation of upregulated circular RNAs after transfection with miR-21, miR-126, or miR-191. This finding may indicate that the formation of circular RNAs by backsplicing occurs most easily in long genes and/or genes divided by a higher number of exons.

## 3. Discussion

Circular RNAs belong to a group of non-coding RNAs with potential regulatory function [8]. In contrast to classical linear transcripts, they form covalently closed loop structures that lack free 3’ and 5’ ends [9]. Circular RNAs were initially thought to be rare isoforms, considered more in the context of potential transcription or folding errors. Instead, it is now known that they occur in abundance and are conserved among species [10]. Dysregulation of circRNA has also been shown to be associated with a wide variety of diseases. The research conducted so far on such relationships has been focused mainly on cancer and neurological diseases [11]. For example, Circ-ITCH is derived from itchy E3 ubiquitin protein ligase (ITCH). Reduced levels of it have been described in bladder cancer sections and correlated with clinical outcomes [12]. The molecule is also degraded in bladder cancer-derived cell lines cultured in vitro. Overexpression of circ-ITCH inhibited cancer cell proliferation, migration, invasion and metastasis. Bioinformatics studies have shown that circ-ITCH can directly sequester miR-17 and miR-224, resulting in increased expression of their target genes *PTEN* and p21, respectively. Additionally, circ-ZFR has been shown to be downregulated in gastric cancer. This molecule regulates GC progression through direct binding to miR-130a/miR-107, in turn affecting *PTEN* expression [13]. Furthermore, they seem to be promising biomarkers [14,15]. These molecules remain stable even under suboptimal storage conditions for biological samples due to their circular structure, which makes them more resistant to degradation by exonucleases. As a result, their half-life in the circulation is longer than that of lncRNAs, in turn likely resulting in slower clearance and higher levels in the circulation. Some studies have shown that the levels of cyclic forms are almost 10 times higher than those of linear forms. Therefore, with optimized assays, they can be detected using basic molecular biology techniques. For this reason, understanding the characteristics of circular RNAs derived, for example, from endothelial cells, as well as the ability to predict potentially functional circRNA molecules, also appears to be important in clinical context.

By analyzing data from endothelial cells with phenotypes similar to those found in healthy individuals, we demonstrated the presence of more than 1000 circular molecules. Most of them have been previously demonstrated and added to public databases, indicating that our data are consistent with previously published studies. In addition, several molecules, including three derived from protein-coding genes, have not been shown before. Of these molecules, circular 13:110213926–110214015, for which the host gene is *COL4A1*, seems particularly interesting. *COL4A1* is a gene located on chromosome 13 that encodes the collagen alpha-1(IV) chain. It is a subunit of type IV collagen that is highly expressed in the subendothelial basement membrane and plays a significant role in many biological processes, such as angiogenesis, among others. Abnormalities in the expression level of this protein have been linked to numerous dysfunctions related to vessel wall integrity, VEGF signaling, and hypoxia, among others. Due to the relatively high sequence variability within this gene, attempts have been made to link *COL4A1* SNPs to cardiovascular or kidney diseases, among other conditions. Therefore, the presence of circular RNA derived from this *COL4A1* gene with potentially high stability in the circulation may be considered a promising biomarker candidate, applicable, for example, in atherosclerosis or other diseases in which angiogenesis disorders predominate. Another interesting finding is 14:90404455–90404514, which is derived from the *CALM1* gene. *CALM1* is one of the genes that encodes calmodulin, which is an important calcium ion-sensitive protein involved in signal transduction. The other genes (*CALM2* and *CALM3)*, although having a slightly different nucleotide structure, encode the same 149-amino acid protein product. These proteins regulate many calcium ion-dependent processes, including the function of cardiac ion channels. Most studies have linked abnormalities within this gene to long QT syndrome. Long QT syndrome is associated with prolonged QT intervals and life-threatening arrhythmias. However, there is also evidence linking single nucleotide polymorphisms within the *CALM1* promoter to osteoarthritis. At the molecular level, this promoter has been linked to effects on the expression of the *COL2A1* and *AGC1* genes and thus on the extracellular matrix. The third circRNA reported is linked to the *GTF2H2* gene. The exact and precise mechanism of action of this gene is not fully understood. Aberrations in its localization are sometimes associated with SMA, most likely due to an associated deletion within the nearby *SMN1* gene. Molecular processes associated with this gene include repair of damaged DNA, which may support the hypothesis of impaired repair mechanisms in the pathogenesis of aneurysmal arterial defects.

Next, we performed a series of analyses with the intent of identifying an approach for the computational analysis of circRNAs that would allow for the prediction of functional RNA molecules as relevant to a given physiological or pathological condition. As the first step, we identified circRNAs with the highest expression levels in endothelial cells, assuming that molecules with high abundance may have functional relevance to a particular tissue. Indeed, of the circular RNAs that were relatively abundant in endothelial cells, several seemed promising candidates for the regulation of endothelial function. In the cells that we used, the circRNA with the highest expression level was hsa_circ_0000284, derived from the *HIPK3* gene. *HIPK3* encodes homeodomain-interacting protein kinase 3, an enzyme that, among other things, participates in the regulation of apoptosis in endothelial cells. In contrast, the circRNA itself, derived from this gene, has already been functionally linked to endothelial cell injury [16]. They showed that circHIPK3 is downregulated during glucose-dependent endothelial injury in diabetic patients. Similar results confirming the potentially functional nature of this circRNA were obtained, demonstrating that exosomal endothelial-derived circHIPK3 promotes proliferation and inhibits apoptosis of vascular smooth muscle cells. This mechanism most likely involves miR-106a-5p, which when trapped by circHIPK3 does not inhibit translation of VCAM pathway proteins [17]. Of the circular RNAs identified in our study, hsa_circ_0001944 showed the highest level of expression among molecules for which the host gene also encoded lncRNAs. The host gene for this molecule is FIRRE, a lncRNA that has been linked to Sotos syndrome, among conditions. The characteristic feature of this syndrome is excessive growth and, as it seems, loss of regulation related to inhibition of growth factors. As a result, the phenotype of patients is complex, but some of them develop cardiovascular disorders, which may confirm the functional endothelial effect of circular RNAs derived from this gene. Furthermore, hsa_circ_0001944 has been linked to growth promotion and metastasis formation in bladder cancer. The level of this circular RNA is significantly upregulated in bladder cancer isolates, and it has been functionally linked to miR-548 blockade [18]. Another potentially functionally interesting circRNA with high expression levels in endothelial cells is hsa_circ_0000523, which is generated from the *METTL3* host gene. *METTL3* encodes a methyltransferase subunit that is responsible for posttranscriptional mRNA methylation. For this purpose, several functions have been demonstrated, such as inhibition of fibrinolysis by promoting PAI-1 expression, as well as mediating inflammatory processes in the vessel wall that promote the development of atherosclerosis [19,20]. For hsa_circ_0000523 potentially functional aspect has been identified, but up to date only for cancer cell line. For example, it was demonstrated that hsa_circ_0000523 regulates proliferation and apoptosis in colorectal cancer cells by interaction with miR-let-7b [21].

The approach involving a comparison of circRNA expression levels with the abundance of linear forms of transcripts derived from the same host gene identified the expression level as higher than that of the linear transcript for 14 circRNAs. The results of this analysis partially agree with the approach of looking for the most abundant circular transcripts; for example, hsa_circ_0000284 or hsa_circ_0002922 are also indicated as potentially functional. However, differences exist, as circular RNAs associated with the *KANSL1* and *KCNN2* genes, among others, were additionally indicated in the first approach. *KANSL1* encodes proteins involved in histone acetylation and has been linked to several cellular processes, including proliferation or mitosis. Mutations within this gene are associated with Koolen–de Vries syndrome, characterized by intellectual disability. The exact function of *KANSL1* in the endothelium is not yet understood, but it has been suggested that, by regulating a number of other genes by the histone acetylation, it is involved in regulation of endothelial cell size and migration [22]. Similarly, abnormalities within the *KCNN2* gene, which encodes a calcium-dependent potassium channel, can be linked to endothelial dysfunction, but the detailed mechanism of the influence that circRNAs derived from these genes have over specific pathobiological states has not yet been demonstrated [23]. However, these examples suggest potential utility of the process of identifying those functional circular RNAs with higher expression levels than the corresponding linear transcript forms.

We also performed an analysis to show whether there is a location in the genome that would preferentially favor circular RNA formation. We compared the number of protein-coding genes to the number of circular RNAs according to a chromosomal location. This approach revealed no significant outliers, and there was a moderate, positive correlation between the number of protein-coding genes with the number of circRNAs by chromosome location. These results are in line with previous findings indicating that circRNAs are expressed along the entire genome.

Subsequently we analyzed the data obtained by stimulating endothelial cells with selected microRNAs, which reproduced a model of altered environmental conditions specific to a common vascular pathology—abdominal aortic aneurysms. In each of the three experiments with microRNA-mediated stimulation, there was a difference in circular RNA levels, and at least a dozen molecules from each condition achieved a statistically significant fold-change in expression levels. These observations are in line with previously published data indicating that microRNA-circRNAs form a complex network of relationships and influence each other’s expression levels [24,25]. Interestingly, we did not observe an overrepresentation of differentially expressed circRNAs derived from the genes directly regulated by our selected microRNAs; rather, the changes were indirect. Moreover, there were four host genes that produce altered expression of circRNAs in every condition associated with microRNA transfection. Reactome databases indicate that these four genes (*RAPGEF5*, *RICTOR*, *PKN1*, *GTDC1*) are associated, among others, with the VEGF-mediated vascular permeability pathway. This finding may indicate that the process of microRNA transfection itself induces a disruption of endothelial integrity, reflected in non-specific activation of related genes, and that the altered expression of circRNAs reflects this process. This finding in turn may suggest that these molecules could act as biomarkers to monitor endothelial homeostasis in a non-invasive manner. However, each of these interactions needs to be investigated separately, as at the global level, we did not observe a clear correlation between circRNA levels and their host genes. In contrast, we were able to show that the greatest potential for altered expression levels of circular RNAs lies with molecules with the ratio of the host gene genomic length to the number of their exons less than the genome-wide average. In the pool of circRNAs identified under stimulatory conditions, we observed overrepresentation of differentially expressed circular RNAs derived from these ‘long intron’ host genes. This finding may indicate that the formation of circular RNAs by backsplicing occurs most easily in long genes and is negatively correlated with the number of exons.

To summarize, in this study, we performed a comprehensive analysis of circular RNAs within endothelial cells. We identified the pool of circular RNAs present in the endothelium and showed their characterization and expression across the genome. Using different computational strategies, we proposed approaches to search for potentially functional molecules. In addition, using data from an in vitro model reproducing conditions in the endothelium from aortal aneurysms, we demonstrated altered expression levels of circRNAs under the influence of microRNAs.

## 4. Materials and Methods

### 4.1. Endothelial Cell Cultures

Cell cultures were performed according to the protocol described in detail in our previous work [26]. Briefly, we used iCell Endothelial Cells (Cellular Dynamics, Madison, WI, USA), which are purified human endothelial cells differentiated from induced pluripotent stem cells, cultured in Vasculite Maintenance Medium (Cellular Dynamics) according to the manufacturer’s protocol. After obtaining confluence of 70–85%, total RNA was isolated using silica-based minicolumns according to the manufacturer’s protocol (TotalRNA Mini, A&A Biotechnology, Gdansk, Poland). The concentration and purity of the RNA eluent were measured by spectrophotometry (NanoDrop Spectrophotometer 2000, NanoDrop Technologies, Wilmington, DE, USA). The quality and integrity of RNA were assessed with Bioanalyzer (PicoRNA Chip, Agilent Technologies, Santa Clara, CA, USA). The resulting RNA was used to prepare libraries preceded by ribodepletion according to the standard Illumina protocol.

### 4.2. Stimulation with Selected microRNAs

A model reproducing conditions in the aneurysmal endothelium was obtained by transfecting endothelial cells with miR-191, miR-126, and miR-21 mimics. Transfection procedures were performed as described previously [26]. Briefly, we used HiPerfect Transfection Reagent (Qiagen, Germantown, MD, USA), which produced complexes with added miRNA mimics (concentration per number of cells). After 12 h of incubation, cells were harvested, RNA was isolated along with them to mock-transfected endothelial cells (described above), and the resulting material was used to create libraries for sequencing.

### 4.3. Library Construction and RNA-Sequencing

Sequenced libraries were constructed using 100 ng of total RNA per sample as starting material, but only high-quality RNA samples (RIN ≥ 9) were used. The first step was performed according to the standard ribosomal depletion protocol from Illumina (San Diego, CA, USA). Sequencing runs were performed with Illumina reagents on a 2500 HiSeq platform (Illumina) at a genomic core facility in 2 × 100 bp in a paired-end manner. The total number of reads for one sample was at least 39,000,000.

### 4.4. Estimation of Selected Circular RNA Expression by Quantitative Real-Time PCR

RNA isolated from endothelial cells was reversibly transcribed using a High-Capacity cDNA Reverse Transcription Kit (ThermoFisher, Branchburg, NJ, USA), according to the standard protocol. Levels of 10 selected circular RNAs were assessed using specific primers and SYBR-Green chemistry. All experiments were performed using a 7900HT Fast Real-Time PCR System (ThermoFisher).

### 4.5. Bioinformatic Pipeline and Statistical Analysis

Raw base call files (.bcl files) were converted to .fastq using the bcl2fastq converter. The FastQC package was used for quality checks. The alignment of paired ends was performed using the STAR package (2.7.0a), which aligned RNA-seq reads to the human genome in a mode that provided correction for spliced junctions. Linear transcript abundance was estimated using the Salmon tool (1.3.0). The DCC package was used to detect and reconstruct circular transcripts. Differential analysis to compare transcriptome under stimulation conditions was executed using the deseq2 R package (1.6.3). Statistical analysis and visualization were performed using R and Prism 7 software, respectively. Statistical significance between categorical variables was assessed using the chi^2^ test. Significance of differences between quantitative variables was assessed using Student’s *t*-test or the Mann–Whitney test, depending on the distribution of variables in the groups. Interdependence was assessed using Spearman’s correlation. A multiple random test procedure was used in the case of large differences between group sizes. The level of statistical significance was set at *p* < 0.05. All the computations were performed on high performance computing systems.

## 5. Conclusions

In the present study, we reported on a comprehensive evaluation of circular RNA in endothelial cells. We determined their species, number, genomic localization of their host genes, and characteristics. Next, using an in vitro model, we demonstrated that stimulation with selected microRNA molecules alters circular RNA levels in a specific manner. We also proposed an index based on the ratio of a genomic sequence length to the exon number of a gene as a useful criterion for selecting pools of circular RNAs with potentially altered expression levels.

## Figures and Tables

**Table 1:** Baseline characteristics of circular RNAs found in endothelial cells.

Feature	Number
Total number of circRNAs	1068
Number of previously annotated circRNAs	1055
Number of circRNAs located within protein coding genes	1007
Number of circRNAs with more than 50 reads	17
Number of circRNAs more abundant than linear mRNA originated from the same gene	14

**Table 2:** List of novel circRNAs originating from protein coding genes.

Coordinates	Host Gene Name	Host Gene Type
14:90404455–90404514	CALM1	protein_coding
5:71035545–71037546	GTF2H2	protein_coding
13:110213926–110214015	COL4A1	protein_coding

**Table 3:** Number of circular RNAs created from one gene. Most genes (more than 500) originate from a single circular RNA, but there are a few providing multiple circular RNAs.

Number of circRNAs Originated	Number of Host Genes
1	587
2	126
3	32
4	12
5	4
6	2
7	3
8	2

**Table 4:** Expression level of selected 10 circular RNAs obtained by RNA sequencing and quantitative real-time PCR. There is a statistically significant, strong correlation between these two measurements.

CircRNA ID	Number of circRNA Counts Obtained from RNAseq [Number of Reads]	Estimated Expression Level Obtained by qPCR [Delta ct between circRNAs and 18S]
has_circ_0000284	210	18.23
has_circ_0000615	67	19.14
has_circ_0001727	61	19.33
has_circ_0008285	54	20.50
has_circ_0001400	39	21.84
has_circ_0001730	35	21.39
has_circ_0001801	27	21.20
has_circ_0000043	11	26.65
has_circ_0001423	7	21.40
has_circ_0004087	6	26.22

**Table 5:** Transfection with microRNA does not cause direct alteration in expression levels of genes which pose as microRNA binding sites.

Stimulant	Number of Differentially Expressed Genes	Number of Differentially Expressed Genes with miR Target Site
miR-191	46	3
miR-126	19	0
miR-21-5p	43	2

**Table 6:** Difference in host gene length to number of exons ratio between stable and overexpressed circular RNAs.

Stimulant	Average Genomic Length to Number of Exons Ratio— Overexpressed circRNAs	Average Genomic Length to Number of Exons Ratio—circRNAs with Stable Level of Expression
miR-191	4277.85	4476.62
miR-126	1207.60	4439.01
miR-21-5p	1559.93	4467.36

## Data Availability

The data that support the findings of this study are available from the corresponding author [S.L.] upon reasonable request.

## Supplementary Materials

The following supporting information can be downloaded at: https://www.mdpi.com/article/10.3390/ijms241210025/s1.
