# Supplementary material for: Comprehensive Analysis of Circular RNAs in Endothelial Cells
Source: Int J Mol Sci. 2023 Jun 12;24(12):10025. doi: 10.3390/ijms241210025 (PMC10298487; doi:10.3390/ijms241210025)
Supplement: Supplementary file 1 [file ijms-24-10025-s001.zip › ijms-2366429-supplementary.pdf]

Supplementary materials for article *Comprehensive analysis of circular RNAs in endothelial cells (Licholai, S.; Studzinska, D.; Plutecka H., Gubała T.,Sanak, M.).*

circBase ID	CircAtlas2 ID	Host Gene Name
hsa_circ_0000284	hsa-HIPK3_0001	HIPK3
NA	hsa-ZNF124_0005	ZNF124
hsa_circ_0002922	hsa-ZNF124_0001	ZNF124
hsa_circ_0001445	hsa-SMARCA5_0005	SMARCA5
hsa_circ_0001829	hsa-SLC45A4_0002	SLC45A4
hsa_circ_0000437	hsa-CORO1C_0003	CORO1C
hsa_circ_0001900	hsa-CAMSAP1_0001	CAMSAP1
hsa_circ_0006156	hsa-FNDC3B_0004	FNDC3B
hsa_circ_0001727	hsa-ZKSCAN1_0001	ZKSCAN1
hsa_circ_0001944	hsa-FIRRE_0001	FIRRE
hsa_circ_0001610	hsa-TNFRSF21_0001	TNFRSF21
hsa_circ_0001681	hsa-RAPGEF5_0006	RAPGEF5
hsa_circ_0000615	hsa-ZNF609_0001	ZNF609
hsa_circ_0001451	hsa-FBXW7_0005	FBXW7
hsa_circ_0000523	hsa-METTL3_0002	METTL3
hsa_circ_0000471	hsa-N4BP2L2_0001	N4BP2L2
NA	hsa-RAPGEF5_0001	RAPGEF5
hsa_circ_0000118	hsa-MAN1A2_0003	MAN1A2
hsa_circ_0008285	hsa-CDYL_0005	CDYL
hsa_circ_0001522	hsa-CSNK1G3_0001	CSNK1G3

Host

Table S1. 20 most abundant circRNAs in endothelial cells

Circular RNA ID	Host Gene Name	Host Gene Description
hsa_circ_0003910	SUMF1	sulfatase modifying factor 1
hsa-C9orf84_0005	NA	not_annotated
hsa_circ_0001947	AFF2	AF4/FMR2 family member 2
hsa_circ_0007375	FBXL13	F-box and leucine rich repeat protein 13
hsa_circ_0001946	LINC00632	long intergenic non-protein coding RNA 632
hsa_circ_0001451	FBXW7	F-box and WD repeat domain containing 7
hsa_circ_0002922	ZNF124	zinc finger protein 124
hsa_circ_0002874	GLIS3	GLIS family zinc finger 3
hsa_circ_0000233	CCDC7	coiled-coil domain containing 7
hsa-RIMS1_0021	RIMS1	regulating synaptic membrane exocytosis 1
hsa_circ_0003380	ZFPM2	zinc finger protein, FOG family member 2
hsa_circ_0000284	HIPK3	homeodomain interacting protein kinase 3
hsa-XKRX_0001	XKRX	XK related, X-linked
hsa_circ_0001947	AFF2	AF4/FMR2 family member 2

Table S2. A list of 14 genes for which the expression level of the linear version of mRNA is lower than that of circular RNA. The Circular RNA ID is given after CircBase, CircAtlas (in that order) of available.
